# Editorial: The Molecular Mechanisms Controlling Sleep Regulation Across Species

**DOI:** 10.3389/fpsyg.2021.702281

**Published:** 2021-06-08

**Authors:** Timo Partonen, Daniela D. Pollak

**Affiliations:** ^1^Department of Public Health and Welfare, National Institute for Health and Welfare (THL), Helsinki, Finland; ^2^Department of Neurophysiology and Neuropharmacology, Medical University of Vienna, Vienna, Austria

**Keywords:** sleep drive, sleep regulation, REM sleep, evolutionarily conserved, chronotype

Sleep is commonly referred to as a period of readily revocable immobility and diminished consciousness during which an organism retains a state of markedly blunted responses to sensory stimuli. This periodic state follows a sleep-wakefulness cycle, and sleep itself is made up of reoccurring cycles of non-REM sleep and REM sleep. Both the circadian and homeostatic processes contribute to the tight regulation of sleep across the animal kingdom, from mammals to birds, amphibians, reptiles, fish and insect albeit the presence, quality, intensity, and quantity of sleep may show significant variability in the course of phylogenetic and ontogenetic development (Siegel, 2008).

Many distinct functions for sleep have been suggested, and profound interactions between sleep and the most important regulatory systems of physiology, including the immune and metabolic systems, have been described, alongside the effects of sleep and deprivation thereof on cellular functions (Allada et al., 2017). Even though there is no definite universal agreement on the precise nature of the evolutionary advantage of sleeping behavior, the preservation of some of the mechanisms regulating sleeping behavior provides evidence for its adaptive value and importance for the integrity for the individual.

This Research Topic on *The Molecular Mechanisms Controlling Sleep Regulation Across Species* comprises a selection of four articles shedding light on different aspects of sleep regulation. Albeit small in number, it is big in volume covering a wide range and providing a lot of information, including the systemic, cellular and molecular levels with relevance for health and disease states in different organisms.

Park and Weber present a Review in which they scope on the neural state underlying rapid eye movement (REM) sleep and its homeostatic regulators. The authors provide a complete overview on the current state of knowledge relating to the neural circuitry and molecular mediators governing REM sleep. Against this background the review article then summarizes and discusses the most important regulators of REM sleep, focusing on the role of sleep pressure (homeostatic regulation) and mathematical models for its physiological integration.

The Mini Review by Yamazaki et al. complements the discourse of Park and Weber by providing an overview of the evolutionary origin of REM and non-REM sleep. The authors comprehensively contrast the current knowledge and view on the principles and functions of non-REM and REM sleep in mammals and birds against the behavioral states of sleep in reptiles, fish and invertebrates. The relevance of this kind of approach with models for tracing the evolutionary development of different sleep states, their functions and molecular genetic principles is being discussed.

In their Review, Ode and Ueda have the focus on emerging evidence for an important regulatory role of synaptic protein phosphorylation as the molecular entity associated with sleep need. They first introduce the two-process model to illustrate the interaction between the circadian and homeostatic principles in the regulation of sleep. This leads the readers to a presentation of a comprehensive elaboration on phosphorylation-dependent mechanisms as a key molecular pathway regulating sleep drive and, reversely, the importance of sleep in the control protein activity.

Intriguing support for a link between the subjective sleep quality and chronotype is presented by Weiss et al. in their Original Research. It provides evidence for an association of circadian gene variants with sleep and mental health. Specifically, they focus on genotypes associated with the *PER3* variable number tandem repeat (VNTR) polymorphism, and they examine the interrelationships with depressive symptoms, subjective sleep disturbances, chronotype relying on the self-reports of sleep schedule, as well as with accelerometer-based estimates of sleep quality and sleep structure. From their study on young adults, the authors report that the *PER3* VNTR genotype predicts depressive symptoms with objective sleep parameters, but not with chronotype or subjective sleep disturbances. However, in men but not in women, there were independent associations of the *PER3* VNTR genotype with depressive symptoms as well as with chronotype. Thus, the *PER3* genotype may provide a shared genetic basis for the aforementioned findings.

The selection of articles contained in this Research Topic touches on some of the most important aspects related to *The Molecular Mechanisms Controlling Sleep Regulation Across Species* emphasizing not only the critical importance of sleep from phylogenetic and ontogenetic perspectives, but also pointing toward the manifold open questions to be addressed in future lines of research.

## Author Contributions

TP and DDP have jointly written the article.

## Conflict of Interest

The authors declare that the research was conducted in the absence of any commercial or financial relationships that could be construed as a potential conflict of interest.
